# Seven-day, free-living, objectively-measured ambulatory activity: a data set from the Dikgale Health and Demographic Surveillance System site, Limpopo Province, South Africa

**DOI:** 10.1186/s13104-023-06547-0

**Published:** 2023-10-19

**Authors:** Ian Cook

**Affiliations:** Physical Activity Epidemiology Laboratory (EDST), University of Limpopo (Turfloop), Sovenga, Limpopo Province South Africa

**Keywords:** Pedometer, Accelerometer, Physical activity, Rural, Africa

## Abstract

**Objectives:**

To investigate first, the level, distribution, patterns and prevalence of seven-day, objectively-measured physical activity (ambulation) in a rural health and demographic surveillance system (HDSS) site in South Africa, across demographic, temporal and anthropometric measures, within a sample of adolescent and adult participants from a defined ethnic group. Second, to investigate the strength and direction of association between levels of adiposity and physical activity (ambulation).

**Data description:**

The data collected comprises anonymized, individual-level, seven-day pedometry data from a cross-sectional, conveniently sampled survey conducted in 2005–2007. The data includes daily steps and daily activity energy expenditure, basic demographic and temporal information (age, sex, village, day, season) and anthropometric measures (stature, body mass, waist and hip circumference, skinfold thickness) and resting heart rate and blood pressure. Given that this data set was of the first large-scale surveys of objectively-measured physical activity in a South African sample, it could be useful for inclusion in future ecological studies investigating the trend of physical activity over time in the South African population. In addition, this objectively-measured data could provide a useful triangulation point for the interpretation and validation of surveys conducted using self-report measures, especially within rural communities.

## Objective

The quantification of physical activity in rural South African settings prior to 2000, was primarily conducted using self-report measures [1, 2]. The use of objective measures of physical activity in especially rural South African communities has shown a significant increase over the last two decades, providing important insights obscured by biases and measurement errors inherent in self-report measures [3, 4]. The objective of this data note is to describe a pioneering data set containing seven-day pedometry output and associated demographic, temporal, anthropometric and blood pressure data (n = 789) collected in a rural South African HDSS site during the period December 2005 to December 2007. This was the first large data set of objectively-measured physical activity collected in a South African HDSS site. In studies published in 2010 - 2012 [3, 5, 6], where this dataset was used, it was found that, compared with results from earlier self-report surveys, objectively-measured physical activity was substantively different. Ambulation levels were high, likely because of a largely subsistence lifestyle within this community. Finally, step data from African settings requires reporting in formats which make it possible to include the results in systematic reviews and thus reduce bias toward data collected from more industrialized settings [5, 6].

## Data description

The data set contains waist-worn, accelerometry-based pedometry data from 789 participants, collected over a period of seven days in a free-living environment. The participants were conveniently recruited into a cross-sectional survey over a two-year period (2005–2007). The participants are resident in various rural village communities covered by the Dikgale HDSS site in the Limpopo Province, South Africa [7]. In order to standardize the sample to the age and sex structure of the larger surveillance site population and other population structures during analysis, age-sex weightings were calculated.

The data set is stored at Figshare [8] in three files, containing the same information using different formats. The first file is a SPSS data file (Pedometer_Dataset.sav, Table 1, Data set 1), the next file is a Stata data file (Pedometer_Dataset.dta, Table 1, Data set 2), and the third data file is a comma-separated file (Pedometer_Dataset.csv, Table 1, Data set 3). Both the SPSS and Stata data files contain variable descriptions and value labels. If the comma-separated data file is used, a comma-separated file containing variable names, variable descriptions and value labels is provided (Variables_DescriptiveLabels_ValueLabels.csv, Table 1, Data file 1). The last data file is an Excel file containing the weighting of the pedometer sample against the Dikgale HDSS and other standard populations (Weighting_2.xls, Table 1, Data file 2).

After permission was obtained from the local community chiefs and leaders to conduct the survey, participants were visited twice over a nine-day period. Participants were from both sexes (female = 516, male = 273), across seven villages, and over a wide age range (14–96 years).

At the commencement of the data collection period, participants provided informed consent, and trained, local fieldworkers conducted interviews, collected anthropometric and resting blood pressure data, and instructed participants on the required procedures for wearing the pedometer. The second visit occurred nine days later to collect the pedometers.

Standard anthropometric measures of stature, body mass, waist and hip circumference, and skinfold thicknesses were obtained. Obesity was defined by body mass index and sex-specific waist circumference cut-points. Resting heart and diastolic and systolic blood pressure were measured after a 10-minute seated rest, using an automated electronic blood pressure monitor.

Piezo-electric pedometers were used to collect seven full days of ambulation data. The waist-worn pedometers were placed on the righ-thand side, and securely attached to a nylon belt. The pedometers could be removed for sleeping and bathing activities.

Ambulation physical activity volume was defined as the average steps/day for the seven days, and for weekdays (five-days) and weekend days (two-day period). Public health step indices (steps/day) were used to classify participants as sedentary, low active, somewhat active, active and very active. Participants were classified as achieving public health pedometry guidelines (≥ 10 000 steps/day) or not. Step classification indices were constructed for each day and for averaged steps. Using a proprietary algorithm, the daily activity energy expenditure was also recorded, and is a function of both the volume (time) and intensity (accelerations) of the recorded vertical body movement.

**Table 1:** Overview of data files/data sets

Label	Name of data file/data set	File types (file extension)	Data repository and identifier (DOI or accession number)
Data set 1	Pedometer_Dataset.sav	SPSS data (.sav)	Figshare (10.6084/m9.figshare.22650964.v6) [8]
Data set 2	Pedometer_Dataset.dta	Stata data (.dta)	Figshare (10.6084/m9.figshare.22650964.v6) [8]
Data set 3	Pedometer_Dataset.csv	Comma separated values (.csv)	Figshare (10.6084/m9.figshare.22650964.v6) [8]
Data file 1	Variables_DescriptiveLabels_ValueLabels.csv	Comma separated values (.csv)	Figshare (10.6084/m9.figshare.22650964.v6) [8]
Data file 2	Weighting_2.xls	MS Excel spreadsheet (.xls)	Figshare (10.6084/m9.figshare.22650964.v6) [8]

Direct URL to data: https://figshare.com/articles/dataset/Pedometer_Dataset_sav/22650964

## Limitations

- The major limitation of these data are that the participants were conveniently recruited. An attempt to address this limitation somewhat was through weighting.
- Due to work-related travel and migrancy, fewer males were recruited than was planned.
- Although the step and activity energy expenditure data were collected through piezo-electric pedometers, as opposed to self-report methods, the technology is dated compared with more recent objective monitoring methods.

## Data Availability

The data described in this Data Note can be freely and openly accessed on Figshare under 10.6084/m9.figshare.22650964. Please see Table 1 and references [8] for details and links to the data.

## Abbreviations

HDSS: Health and Demographic Surveillance System
